# Behavioural valuation of landscapes using movement data

**DOI:** 10.1098/rstb.2018.0046

**Published:** 2019-07-29

**Authors:** George Wittemyer, Joseph M. Northrup, Guillaume Bastille-Rousseau

**Affiliations:** 1 Department of Fish, Wildlife, and Conservation Biology, Colorado State University, Fort Collins, CO 80523, USA; 2 Wildlife Research and Monitoring Section, Ontario Ministry of Natural Resources and Forestry, Peterborough, Ontario, Canada K9J 8M5; 3 Environmental and Life Sciences Graduate Program, Trent University, Peterborough, Ontario, Canada

**Keywords:** home range, migration, biologging, resource selection, optimization, landscape conservation

## Abstract

Wildlife tracking is one of the most frequently employed approaches to monitor and study wildlife populations. To date, the application of tracking data to applied objectives has focused largely on the intensity of use by an animal in a location or the type of habitat. While this has provided valuable insights and advanced spatial wildlife management, such interpretation of tracking data does not capture the complexity of spatio-temporal processes inherent to animal behaviour and represented in the movement path. Here, we discuss current and emerging approaches to estimate the behavioural value of spatial locations using movement data, focusing on the nexus of conservation behaviour and movement ecology that can amplify the application of animal tracking research to contemporary conservation challenges. We highlight the importance of applying behavioural ecological approaches to the analysis of tracking data and discuss the utility of comparative approaches, optimization theory and economic valuation to gain understanding of movement strategies and gauge population-level processes. First, we discuss innovations in the most fundamental movement-based valuation of landscapes, the intensity of use of a location, namely dissecting temporal dynamics in and means by which to weight the intensity of use. We then expand our discussion to three less common currencies for behavioural valuation of landscapes, namely the assessment of the functional (i.e. what an individual is doing at a location), structural (i.e. how a location relates to use of the broader landscape) and fitness (i.e. the return from using a location) value of a location. Strengthening the behavioural theoretical underpinnings of movement ecology research promises to provide a deeper, mechanistic understanding of animal movement that can lead to unprecedented insights into the interaction between landscapes and animal behaviour and advance the application of movement research to conservation challenges.

This article is part of the theme issue ‘Linking behaviour to dynamics of populations and communities: application of novel approaches in behavioural ecology to conservation’.

## 1. Introduction

Animal movement data, consisting of temporally explicit relocations of individuals in space, provide detailed insights into animal–environment interactions. Because all environments are spatially structured (heterogeneous with respect to the distribution of different biotic and abiotic elements), movement is the fundamental behaviour by which individuals access resources, avoid risks and interface with conspecifics [1]. As such, movements underpin variation in individual fitness and other ecological and evolutionary processes such as gene flow, community structure and species density and distribution [2,3]. The increasing ease of collecting movement data on animals holds immense promise for providing unique understanding of how and why animals move across space, driving a renaissance of research into spatial ecology [4,5]. Critically, a mechanistic understanding of drivers of the movement offers actionable information to address contemporary conservation challenges, with specific application to landscape planning, wildlife protection, mitigating human wildlife conflicts and managing invasive species [6–9].

Technological advances in biologging and other sensing approaches used for animal tracking [9,10] have enabled the collection of fine-scale, long-term relocation data on organisms, which provides new opportunities for both basic (e.g. ontological changes in strategies) and applied (e.g. a response to human-driven landscape changes) research. With these advances, the quality and complexity of movement data has increased exponentially [10]. Given that most data on movement are captured remotely, common constraints of observational studies can be avoided (i.e. partial observability, bias and observer influence). However, such data present different challenges related to the contextualization of animal relocation data, specifically the interpretation of why an animal uses a given location without having adequate information on the latent process of interest.

Approaches used to study animal behaviour provide a useful lens through which to view movement ecology research that can lead to a deeper understanding of movement processes. Building on the fundamental currency of ecological and evolutionary research on behaviour, a key direction in movement ecology is to develop links between spatio-temporal behavioural patterns and metrics of fitness (e.g. resource aggregation rates, survival or reproduction) to enhance understanding of the causes and consequences of movement behaviour [11]. Classically, behavioural studies of this nature focused on quantifying fitness payoffs (i.e. the value) of behaviours of interest and contrasting the returns from different strategies (i.e. optimality analysis). However, the accurate quantification of the collective costs and benefits of animal movements (i.e. derivation of remotely sensed metrics of fitness to relate to movement decisions) is challenging given they are influenced by highly variable intrinsic and extrinsic factors [12]. As a result, research has focused more commonly on the consequences of movement to population distribution [2] or the description of movement phenomena [13]. Recent applications of tracking-based monitoring have allowed the quantification of fitness parameters, allowing insights to the relation between fitness and movement tactics (e.g. reproductive rate [14] or mortality [15]) or lack thereof [16]. Developing and quantifying individual location-based metrics derived from movement that accurately capture mechanistic and functional aspects of key behaviours can offer a powerful direction to advance movement ecological research, connect it explicitly to its behavioural theoretical underpinnings and, thereby, enable direct application to conservation challenges.

**Figure 1.**
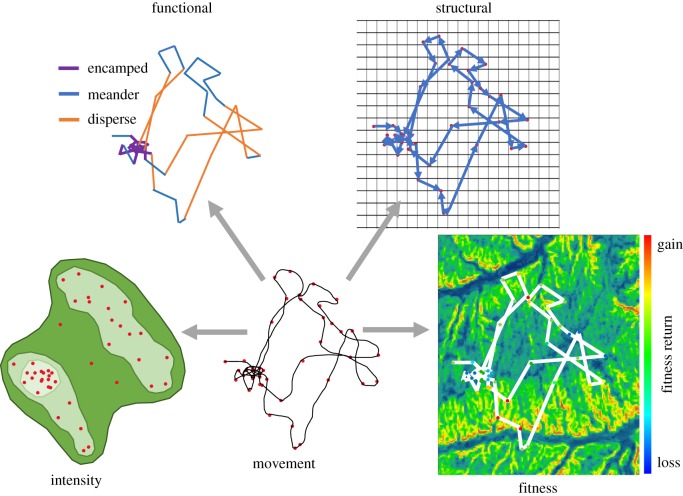
The movement path of an animal, sampled periodically using GPS telemetry, offers rich information on animal behaviour. To facilitate greater use of these data, we outline four approaches to estimate the behavioural value of spatial locations based on movement data. These approaches include the assessment of the *intensity* of use (e.g. density isopleths), *functional* use (e.g. movement states), *structural* aspects of use (e.g. network graphs) and *fitness* values of locations (e.g. energetic maps) as detailed in table 1.

Because movement data are inherently spatial and the behaviours they represent have direct links to fitness, one of the most exciting promises of movement ecology is the ability to interpret the value of landscapes to individuals. However, this concept has been underserved in the literature. Here, we aim to consolidate the current approaches used to value locations within landscapes from movement data. By providing a template for developing indices capturing different aspects of movement, we intend to facilitate understanding of the importance of a location to an individual. We aim for this compilation to serve as a framework by which to interpret movement through its fundamental behavioural underpinnings and, thereby, help consolidate the mechanistic and theoretical foundation of the discipline of movement ecology. To enable a behavioural ecological approach to movement analysis, we first highlight relevant topics at the interface of behavioural theory and movement ecology. We then outline and discuss four fundamental currencies for the economic valuation of landscapes using movement data (table 1). Initially, we review the classic movement ecological currency focused on valuing locations by quantifying the intensity of use, highlighting the importance of using approaches that explore heterogeneity (including temporal dynamics) in this process. We then discuss three additional approaches for valuing locations from movement data, each of which offers unique insights into an aspect of movement behaviour: interpretation of the function of movement, assessment of the structural properties of locations relative to the broader landscape and commodifying movement data in a fitness framework (figure 1). We highlight key areas where these approaches can be developed further that will provide greater insights into animal behaviour and locational importance. Finally, we discuss the implications for our analyses to conservation.

**Table 1.** Classes of movement metrics for defining the behavioural value of landscapes depicted in figure 1.

class	definition	metrics	methods
*intensity*	How much is a location used?	fix density, time density, weighted use, persistence velocity, dot product, time to return, first passage time, probability of occurrence	home range estimation, habitat preference, resource selection, recurrence
*functional*	What is an individual doing at a location?	speed, movement states (based on turning angle and speed)	hidden Markov modelling, behavioural change point analysis, agent-based models
*structural*	How does a location influence use of the broader landscape?	connectivity, proximity, neighbourhood statistics (degree, interspersion, isolation, dispersion), network metrics (weight, degree, centrality)	network theoretical approaches, circuit theory, Fragstats, least-cost path
*fitness*	What is the payoff of a location?	caloric expenditure/return, reproduction, survival, risk (predation), fitness proxies	physiological modelling (basal metabolic rate), vaginal implant transmitters, mortality monitoring, overall dynamic body acceleration

## 2. Behavioural underpinnings of movement data

The key approaches to research design common to behavioural ecological research are well suited to movement ecology and can provide useful direction to the analysis of movement data. Prominent advances in behavioural ecological theory have been derived from the use of a comparative approach, economic valuation of behaviours and assessment of optimality [17]. Movement data are ideal for such approaches given they provide repeated measures on an individual, which is the fundamental unit of data collection in behavioural ecology. Furthermore, the spatio-temporal nature of the data allows the resolution of fundamental units of behaviour with conservation relevance (e.g. time-spent, expenditure and relative use of locations).

While the preponderance of the movement ecology literature has focused on method development to account for the complex spatio-temporal structure inherent in the movement process, the basic tenets of movement ecology are underlain by fundamental ecological and behavioural theories. The behavioural theory with the most direct application to the study of animal movement and space use is optimal foraging theory (i.e. patch/diet selection, marginal value theorem (MVT), giving up densities and landscape of fear), given movement has served as a unit of valuation to assess foraging strategies [18]. For instance, the MVT [19] provides the framework for patch-occupancy dynamics, formulated in respect to the time spent in a location rather than the nuances of the movement in a location. Relatedly, the theory of ideal free distribution (IFD) largely underpins the interpretation of resource selection behaviour [20,21]. While these foundational theories have served as a critical springboard to understanding movement behaviour, their application to systems that lack the basic metrics upon which these theories are based (i.e. energetic gain in the case of MVT and population growth in the case of IFD) merits consideration. For example, while the IFD fundamentally underpins our interpretation of habitat selection (i.e. intensity of use reflects differences in available resource value), intraspecific competition can lead to opposite mechanisms driving the intensity of use (i.e. density of use may reflect refuge habitats from despots rather than the intrinsic fitness value of the landscape) [21]. Similarly, incorporation of perceived risk can fundamentally structure distributions and use of habitats [22,23], which again can lead to misinterpretation of the value of specific locations on the landscape based solely on the intensity of use behaviour. Despite wide recognition of these drivers of the underlying process structuring space use, the risk of misinterpreting the mechanism underlying the intensity of use can be minimized through study design or with supporting behavioural information.

Despite the dominant role of foraging theory in structuring the analyses and questions asked of movement data, integrating movement ecology with other behavioural theories promises to enhance a practical understanding of animal space use as well as advance the testing and development of behavioural theory across larger scales and a greater number of individuals than can be accomplished with traditional methods such as direct observation. We highlight several fundamental behavioural lines of inquiry that movement is uniquely suited to help resolve (table 2). With access to increasingly high-resolution data (both spatially and temporally), greater insights into drivers of differentiation in the intensity of use are being derived by applying approaches initially used to study behavioural and phenotypic plasticity. Specifically, quantifying reaction norms (i.e. the patterns of individual phenotypes or behaviours expressed across an environmental range [32], analogous to functional responses when assessed at the population level [33]) in respect to resource selection along gradients of landscape variables is a particularly powerful analytical framework to understand individual responses to and requirements of landscape features. This framework is particularly valuable for exploring behavioural expression related to changes in landscapes, as commonly induced by human activities [34].

**Table 2.** Measuring costs and benefits of key behaviours using movement

behavioural category	example issue	example metric	reference
optimal foraging theory	search behaviour	patch occupancy	[24]
trade-offs	dispersal decisions	net squared displacement	[25]
competition	costs to subordinates	rank-based space use	[26]
alternative strategies	male reproductive states	daily activity budget	[27]
group living	group size constraints	daily movement distance	[28]
parental investment	impact of maternal investment on survival	foraging trip length	[29]
behavioural syndromes	factors influencing different movement tactics	resource selection	[30]
plasticity	response to human activity	vagility	[8]
seasonal response	influence of longitudinal changes in resources	behavioural change point analysis	[31]

Theoretically underpinned behavioural metrics derived in a spatially explicit manner can be interpreted as the value of landscapes for those behaviours. Linking behaviour to spatial locations provides a relevant and tangible means by which to summarize behavioural responses to landscape features, which can provide an elemental insight for structuring conservation planning and actions spatially. From the fore-mentioned functional response analyses that help elucidate behavioural modification of space use across gradients of particular features [35] to the quantification of the spatial degree of feature avoidance that can be translated into estimates of habitat loss [36], better resolution of spatial behaviour relative to landscape features has direct applied relevance. Mechanistic underpinnings of the interpretation of a metric are particularly important for the application of movement ecology to conservation objectives. A further development of metrics with strong theoretical underpinnings are important to resolve the specific contexts in which such metrics are meaningful.

## 3. Dissecting heterogeneity in the intensity of use

The focus of many movement-based analyses centres on the intensity of use metrics to infer the value of locations within landscapes and the theoretical underpinnings of such procedures are well established. The analysis of time spent in given locations can provide insights into investment in those locations by animals, which is a critical component of fitness. However, the interpretation of time spent in a location can be complex, and assumptions risk misinterpreting the rationale for prolonged use of a location (e.g. territorial exclusion from preferred areas *sensu* predictions from ideal despotic distribution theory). Awareness of this challenge has driven efforts to restructure the analysis of the intensity of use metrics. While we discuss the value of differentiating the behaviours associated with recorded locations when analysing movement data below, here we highlight the importance of using approaches to investigate spatio-temporal heterogeneity in the intensity of use across and within individuals. Behavioural assessment at the individual level is critical to resolve underlying mechanisms of the intensity of use patterns. While still the norm, simple aggregation of movement data to determine the intensity of use underserves the rich spatio-temporal information being collected through GPS tracking on movement patterns of individuals [37]. Direct assessment of the temporal aspect of space use, as outlined by time geography (see below), offers insights into heterogeneity in space use by an individual. In addition, we discuss the value of focusing analyses at the unit of data collection, the individual, rather than aggregating across individuals to derive an average, population-level metric.

**Figure 2.**
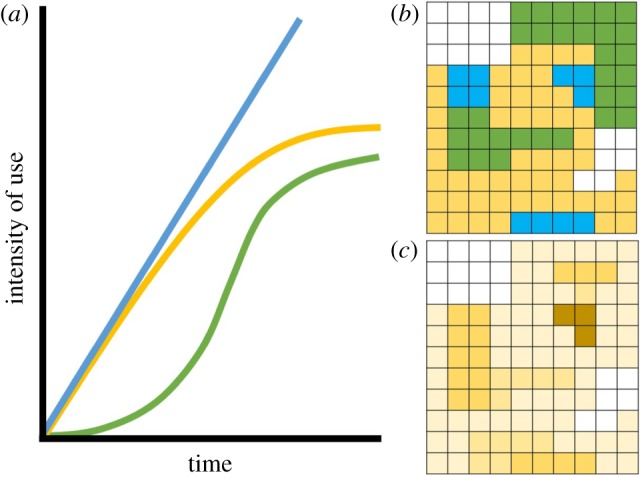
(*a*) Mirroring functional response in predator foraging behaviour relative to prey density, the intensity of use of specific locations can be assessed in terms of differential temporal patterns. Type I use indicates a consistently used location, such as a den site or water point in an arid environment (blue). Type II use indicates a location where use saturates, such as at point resources that experience denudation with increased use (yellow). Type III use indicates temporally sporadic use, such as seasonal resources that are available intermittently and are denuded quickly (green). (*b*) Plotting different functional use types on the landscape can elucidate differences in the intensity of use patterns. (*c*) Contrasting with raw intensity of use data (darker indicates more use) can discern not only how much an area is used, but also the structure in temporal use patterns.

Time geography is an integrative framework focused on the analysis of both the spatial and temporal dimension of a process [38]. Temporal variation in use can serve to identify changes in landscape value driven by ecological dynamics (i.e. seasonality) or related to known ontogeny (i.e. life-history stages), serving to address questions focused on the underlying biological dynamics in a system (table 2). The intensity of use of a location can be assessed as a function of time, analogous to the classic functional response where the intake rate of a consumer is a function of food density [39]. Such an approach (i.e. plotting cumulative use as a function of time) can distinguish a consistent increase in use intensity (type 1) reflecting daily required use (e.g. water hole), from use that saturates at a point in time (type 2) as expected to result from resource denudation, or sporadic use of a location (type 3) associated with resource pulses. These patterns of use can then be plotted to offer insights into heterogeneity in behaviour (figure 2). While relatively infrequent in the literature, one of the most interesting aspects of temporal dynamics in movement are return rates or revisitation. Revisitation and directed interpatch movement coupled with short-term occupancy may indicate strikingly a different value of a location than an extended, non-repeated stay [40,41], but would be considered equally in most contemporary analysis of the intensity of use [42]. The analysis of recursion rates or time spent in a patch initially focused on herbivore–vegetation dynamics, with relevance to range management [43], which has provided new insights to herbivore ecology, migratory behaviours and interspecific interactions [42,44]. Building on this, recursion and directed movement are recognized as potentially key behaviours to facilitate the study of cognitive decision processes and spatial memory [45,46]. Longer-term repeated-use patterns, such as inter-annual [47] or inter-generational [48] migratory patterns, offer additional avenues to investigate responses to slower landscape dynamics (e.g. phenological shifts and land-use change). Contemporary work focused on refined definitions of spatial and temporal patterns of use can provide new insights into the behaviours underlying the movement process.

While recursion and other temporal patterns of use are an important type of heterogeneity in the intensity of use metrics, the heterogeneity of use between time periods by an individual (i.e. ontogeny) or across individuals is another critical facet that can serve to gain understanding of behavioural mechanisms. Movement studies frequently smooth over the heterogeneity among individuals, time period (e.g. years) or both, reducing the inference gained by analyses of movement data. Normative behaviours are commonly used to derive landscape-level assessments, either through the density of use metrics or weighted approaches such as resource selection functions, which take into account the relative availability of a location attributes when analysing its weight of use. However, assessing and representing heterogeneity across individuals is critical for accurate characterization of a locational value based on use intensity, particularly given that heterogeneity can be driven by variation in responses to features related to personality, different life-history strategies (i.e. behavioural tactics) or different experiences (e.g. learned knowledge base) [46]. Leveraging variation in individual spatial behaviour in a comparative analytical approach common to behavioural ecological studies can help resolve the underlying mechanisms of heterogeneity. Such an approach can lead to a refined understanding of differences in spatial behaviours (as manifested from different tactics) or requirements (e.g. clustering individual-level resource selection function (RSF) coefficients to define tactics in a population [30]). Furthermore, juxtaposing the intensity of use between conspecifics can provide deeper insights into a landscape value, as exemplified by studies of ecological traps that identify features serving as sinks [22].

## 4. Assessing the function of movements

**Figure 3.**
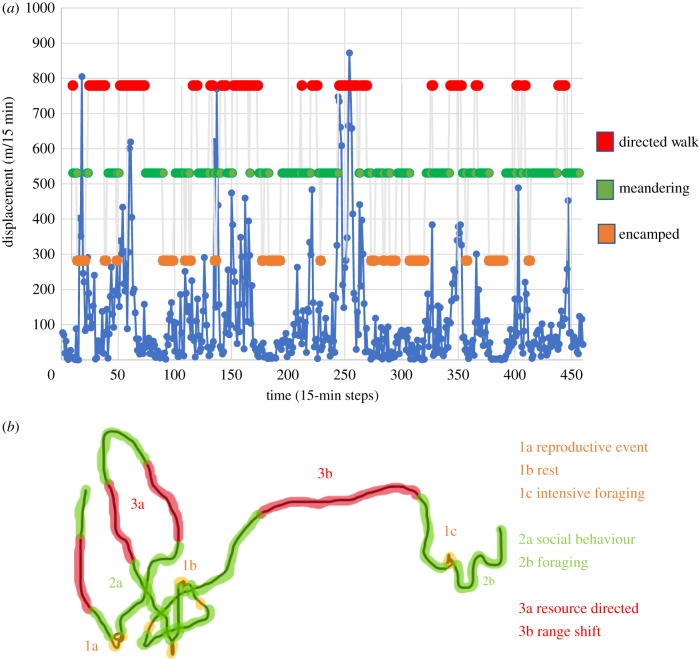
Discretizing the movement path of an individual can elucidate structure in movement behaviour. (*a*) Plotting the step lengths shows heterogeneity in speed often equated to different behavioural functions of the animal's motion (blue line). Similarly, heterogeneity in turning angle captures aspects of the behavioural function of the animal's movement (not shown). Using approaches to identify probabilistic-based movement states allows the simplification of the movement into specific categories of motion (e.g. directed walks characterized by high speed and little change in bearing (red), meandering characterized by slower speed and less direction (green), and encamped characterized by short to no displacement and little directional persistence (orange)). (*b*) Overlaying the state definition of the movement path helps elucidate structure in the movement path. Relating these defined states to observed behaviour can resolve the function of the movements.

Determining the function of movements (i.e. what animals are doing at a location) remains a fundamental focus of movement ecology, as it provides a critical lens by which to understand behaviour, interpret the intensity of use metrics and evaluate locations on the landscape behaviourally [1]. Broadly, functional identification of movement typically focuses on mechanic definition (i.e. properties or type) or phenomenological characterization (i.e. underlying motivation) of a movement segment. In the former, the movement path or locational use can be discretized into movement states (e.g. encamped [49]), while in the latter different behavioural phenomenon, often identified independently, can be used to stratify the movement path (e.g. periods of rest [50]; figure 3). Complicating such functional definition is the fact that characteristic movement properties can reflect multiple behavioural phenomena (i.e. encamped movement state may reflect resting, nesting or intensive foraging).

Common approaches to discern the function of movements using the mechanics of a path define structural transition points (i.e. changes in movement properties) that serve to categorize movement types/states from which its function can be discerned. The definition of movement properties along the path can be used for discretization, whereby modelling approaches are used to categorize observations (e.g. locations) into putative states (figure 3*a*). This approach provides insights into latent processes and has become more common in the movement ecology literature [31,51,52]. Resolving the type of movement represented at different locations and the frequency with which such movements occur provides critical insights into activity budgets, attractants and feature avoidance, which ultimately allow inference to movement function. Such approaches have the potential to identify when and where the motivation for movements shift. This inference can be gained directly by simple density functions of movement states [53] whereby types of movement are related to landscape features, by contrasting the timing and relationship between movement states and known features [45] or using relative approaches such as RSFs or step selection functions on each state independently or in combination [54]. Such approaches provide a means to resolve mechanistic drivers of spatial heterogeneity in occurrence and population distribution building on approaches highlighted above (see §3). Analyses of discretized movement paths have also provided rare insights into the function of memory in movement behaviour, one of the fundamental and enigmatic areas of movement ecology [46]. Despite the importance of determining its underlying function, and the valuable insight gained by doing this, relying on the movement path itself to define function, however, is challenging without contextual information [52].

Phenomenological determination of movement properties provides an alternative approach to investigate movement function. Typically, phenomenological characterization entails additional information paired with the movement data, including data from paired sensors (e.g. physiological biologgers) or observations (e.g. reproductive condition) that are then used to isolate movement segments relative to a behavioural phenomenon of interest (figure 3*b*). Structuring analyses in a comparative framework to contrast movement properties across known behaviours can provide insights into different space-use properties and degrees of investment associated with specific behaviours [26]. Coupled behavioural monitoring, via direct observation or sensors, with movement has the potential to provide some of the most powerful inference on the movement process and promises to be a primary means by which to resolve function, including the influence of social and reproductive behaviour, physiology, perception (e.g. risk aversion) and ecological drivers (e.g. insect, parasite/disease and climate) on movement [55,56]. Technological innovations allowing new sensors to be coupled with tracking have enabled a diversity of new approaches to define underlying phenomena and interpret movement function [57]. Most notably, accelerometery has served as a key instrument to resolve activity levels related to locational positions and, in some cases, provides insights into energetic balance [55]. Building from this, additional sensors that record or allow a direct observation of the state and behaviour of the individual (e.g. animal borne video, acoustic, magnetometers and physiological monitoring systems) as well as sense conditions experienced in the environment (e.g. temperature, salinity and humidity) are opening novel avenues to resolve the drivers and function of movement [58].

While analytical approaches in isolation or relative to landscape features have provided important insights, multiple functions can be reflected in the same or similar movement path characteristics (or sensor measurements) (figure 3*b*). For instance, when using movement to resolve foraging behaviour, different resource distributions can elicit strongly differentiated foraging movements (e.g. high-value clumped versus dispersed resources can result in encamped or meandering movement paths), limiting the efficacy of describing a foraging state directly from the movement path. Given these limitations, coupled sources of information can provide confidence in interpretation or facilitate the quantification of uncertainty in functional definitions [59].

Understanding the functional driver of movements is fundamental to developing understanding of species distributions. Such insights can be used to demarcate habitats critical for survival and reproduction (e.g. important foraging grounds) or the spatial requirements for certain life stages (e.g. nesting or denning sites), information critical for species-specific landscape planning. Classically, the intensity of use metrics have largely been interpreted functionally, but improved resolution of movement data collection coupled with sensor or observational data provides elemental insights into the actual function of movements at different locations. Such combined data threads enable an improved valuation of landscapes and thus targeted spatial conservation planning with fewer assumptions [60].

## 5. Understanding the structure of the landscape

**Figure 4.**
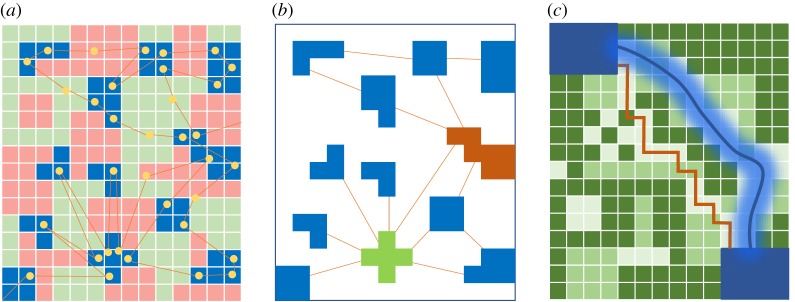
Structural valuation is based on the importance of a location for the broader landscape context. (*a*) An animal's movement crosses over different resources on the landscape. (*b*) Discretizing a landscape into patches (using resource patches or movement properties) can be used to portray the landscape as a matrix. Quantifying connections among patches can be used to derive network metrics—the green patch has a high degree centrality value (key landscape hub) and the orange patch has a high betweenness centrality value (key bottleneck in the network). (*c*) Resistance surface maps evaluate the cost for animal movement with the darker green representing a higher cost. Optimization approaches highlight different features of the landscape, here portrayed by the orange line representing the movement corridor linking the two blue patches based on a least-cost path approach and the blue line represents an estimation of a likely corridor estimated based on the circuit theory.

Defining a location by its structural role in the larger landscape as defined by an individual's movement provides an intermediate approach to the classic Eulerian (focusing on emerging population patterns) and Lagrangian (focusing on individual's movement steps) paradigms for characterizing movement [4,61]. We term this category structural valuation, whereby locational importance emerges from the role or function it plays within overall movement trajectories across the landscape [62]. Structural valuation of landscapes is related to work focusing on landscape ecology and movement connectivity, increasingly involving network- (or graph-) theoretical approaches (figure 4) or least-cost path approaches [63]. When applied to movement, network theory discretizes animal space use into different locations or patches on the landscape (referred to as nodes) and the potential connections (edges or links) among these locations [62,64]. Network metrics can also serve to characterize the importance of locations on the landscape in terms of use, connectedness and centrality measures [65].

Movement data have improved the application of network theory to spatial ecology in three ways that formerly relied on expert opinion-based definitions: definition of patches, quantifying connectivity and characterizing movement cost. Focusing on patch definition (figure 4*a*), structural valuation of landscapes can provide parallel inference to that derived from metrics of the intensity of use [66]. This allows the quantitative definition of preferred landcover types [67] or specific locations of behaviours of interest (e.g. central-place foraging or reproductive sites) [50]. In addition, centrality measures (e.g. degree and betweenness) can identify important areas even if animals do not spend a disproportionate amount of time there (figure 4*b*), as can emerge when cognitive processes drive directed long-distance movement [46]. Despite the low intensity of the use of corridors connecting chosen locations, their importance can be high if their disappearance prevented animals from accessing a specific area [65]. By defining the movement landscape in terms of patches (nodes) and connectivity corridors (key edges), movement summarization using network theory provides a natural structure for investigating patch-occupancy dynamics and testing ecological theory (e.g. the IFD or marginal value theorem) or exploring alternative processes affecting connectivity [68].

Combining movement networks with spatial environmental data can facilitate patch and movement resistance characterization across broad landscapes (i.e. the suitability of the landscape to movement flow) (figure 4*c*). The definition of patches and resistant surfaces can then be used to estimate optimal linkages (corridors) among patches using least-cost path, circuit theory and ‘hybrid’ approaches such as randomized shortest path [69]. Pros and cons of the different algorithms and how to best use these approaches have been the subject of extensive discussion, including that such optimality approaches may be misleading given wildlife often traverse unsuitable habitat or avoid apparently suitable habitat for alternate reasons [68,70,71].

Validation of movement networks is rarely done but is important for ensuring usefulness for conservation [72,73], as there is a potential risk that areas identified as potential movement corridors or important patches from a connectivity point of view are not actually used by individuals [66,68]. As with all extrapolative modelling, this is particularly the case when predicting beyond the area where data were captured. The validation of network approaches usually requires out-of-sample testing or additional streams of data such as camera or mortality data [66,74].

Where movement data are available for a large fraction of a population, network theory can be applied directly to the tracking data without relying on assumptions regarding how spatial covariates influence space use, patch definition or resistance [62]. For instance, applying network theoretical approaches to a grid overlaid on the movement landscape allows empirical definition of patches and corridors based on emergent properties of the network directly (i.e. assumption-free definition). Most usefully, this can serve to identify indirect connectedness (e.g. circuitous connecting paths). Such an approach avoids misspecification that can occur where subjective means are used to differentiate patches from non-patches or optimality approaches are used to define corridors [62]. Given the reliance on empirical data, outputs from such approaches provide a robust estimation of the structural value of an area, from which unexpected features of the movement landscape can emerge. However, such empirically derived definitions are conditioned on the explicit sample analysed, meaning sample design is critical to the derivation of general conclusions.

## 6. Towards a fitness landscape

**Figure 5.**
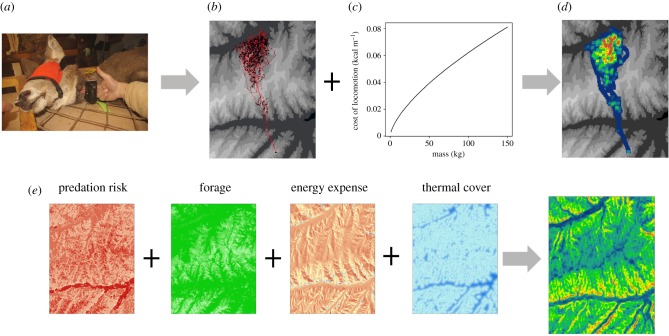
Deriving a fitness landscape from combined movement and landscape data can be achieved using two general approaches. In the upper pathway, individual animals are collared (*a*) and ancillary information (e.g. body condition) is collected. The data provide movement paths (*b*) that can be coupled with information on the metabolic costs of movement (*c*) to produce estimates of the location-specific energetic cost for the animal (*d*), a proxy of the fitness landscape. In the lower pathway (*e*), ancillary (e.g. remote sensing) or modelled movement data are used to create landscape-level layers of fitness components (e.g. predation risk, forage availability, energy expenditure and thermal cover). Aggregation of these layers provides a more comprehensive estimate of the fitness landscape.

Equating individual behavioural strategies to their fitness payoff provides fundamental inference on ecological and evolutionary processes, but collecting the data required for such assessments is challenging in natural systems [75]. The current renaissance in movement ecology allows the simultaneous collection of behavioural data on many individuals over long times. These data often are collected conjointly with fitness proxies such as reproduction, condition or survival, providing a platform for investigation of the links between behaviour and fitness [14,15]. Although movement data have been used to infer links between broadly defined behaviour and fitness [12,76], quantifying the fitness landscape (i.e. spatially explicit predictions of fitness costs and benefits) is a tantalizing prospect that would allow unparalleled insights into the mechanisms underlying the spatial behaviour of animals and, theoretically, allow maximally effective spatial conservation strategies. The ingredients for deriving fitness landscapes seem to be present (i.e. spatial predictions of behavioural metrics that have theoretical links to fitness). However, a number of key challenges exist stemming from the fact that the remote capture of movement data limits inference on the nuances of the behaviours characterized from tracking data and their fitness effects. Developing the opportunities and addressing the limitations of tracking data to facilitate a greater understanding of the links between fitness and behaviour are critical if movement ecology is to provide robust information on ecological and evolutionary dynamics to inform conservation. Deriving a fitness value of the landscape from movement is the most challenging of the four metrics we describe, but also the metric which offers the deepest inference. Broadly, fitness valuation of movement can occur by assigning fitness contributions to specific spatio-temporal events along individual movement paths or the derivation of landscape-level estimates of key fitness components, which can then be related to the movement path (figure 5). Both provide a powerful framework for investigating movement mechanisms and valuable information for addressing conservation and management objectives.

Fundamentally, we rarely have true measures of the fitness of an animal, driving a practical focus on quantifying components of fitness, such as reproduction and survival, or proxies of fitness, such as energetics. At the landscape scale, the focus is often on quantifying features representative of more tangible costs and benefits, such as predation risk, energetic cost of movement and forage availability (e.g. [15]). Commonly, this is done by relating movements to the direct measurement of landscape properties that have fitness relevance such as vegetative or a soil-based nutritional value (e.g. from remotely sensed layers) [77], resistance surfaces (e.g. from topographic features or distance) and abiotic features (e.g. thermal exposure) [78]. Increasingly, model-based estimates of landscape-level fitness surfaces, estimated from movement data, are also being added to such assessments (e.g. the risk landscape); for instance, movement data identify times and locations of reproductive or mortality events, which can be related to environmental factors to derive landscape-level correlates of these key events [16,79,80]. At the individual path scale, high-fidelity activity sensor data (e.g. accelerometers) or physiological monitors integrated into collars are increasingly available. This information can be blended with knowledge on basal metabolic rates and estimates of the increased cost of movement to produce relatively accurate measures of energetic expenditure along the movement path [55]. While derivation of the energetic costs of movement from paired tracking and sensor data are being developed, accessing information on energetic gain or return is more difficult, due to the highly variable temporal dynamics, distribution and quality of different food items. Data on prey capture and consumption rates from individual paths [56] may provide the best opportunities for high-resolution inference on energetic gain. Combining these data with multiple measures of individual animal condition, taken over relatively short-time periods, and the intervening movement data can provide information on the actual gains or losses experienced by the animal [56]. However, this information is difficult to obtain in most systems. Finally, focused analyses of movements around critical events, such as reproduction and mortality events, can serve to derive path-specific valuations [79,81].

While methods for developing the fitness value of landscapes are becoming more accessible, studies doing so are rare and typically quantify a single fitness component and its relationship to coarse representations of movement (e.g. home ranges). Integrating spatial representations of several components or proxies of fitness into a single analysis is beginning to be achievable in some systems (figure 5) and will facilitate more accurate spatial representation (i.e. mapping) of movement relative to the totality of their costs (e.g. mortality) and benefits (e.g. reproductive outputs). Metrics derived from individual movement paths can be considered behavioural strategies, and overlaying these on mapped proxies of fitness can provide inference to the optimization of space-use strategies or trade-offs individuals face when balancing the requirements of multiple contrasting fitness components [82]. Devolving spatially explicit representations of fitness components associated with a given behaviour (i.e. contribution to survival and reproduction) will remain difficult where that fitness contribution is a function of integrated behaviours over space and time. For example, given knowledge on lifetime reproductive success and the lifetime track of an animal (something that is becoming a reality for some species), biometric monitoring or repeated recaptures will be needed to identify location-specific contributions to reproductive success. Regardless of these challenges, focus on accurately quantifying the fitness landscape will drive understanding of the mechanisms underlying movement, individual decisions and population distributions.

## 7. Integrating movement and behavioural ecology to advance applied understanding

Given the foundational role of movement to a diversity of behaviours that influence individual fitness as well as population distribution and community structure, movement ecology underlies numerous disciplines and is fundamental to applied animal conservation. The richness of contemporary movement data collection offers new avenues for individual-based analyses to build deeper insights into fundamental behavioural questions. Extracting informative patterns from the complex structure found in movement data to infer underlying motivations of movement is challenging and has driven a blossoming of analytical advances in the discipline of movement ecology [83]. However, practitioners of movement analyses are now faced with a daunting number of approaches, often to estimate a single process (e.g. home range estimation), with cursory integration with theoretical underpinnings. Despite burgeoning methodological development, the insights gained through such approaches are constrained, and understanding those constraints is critical to effectively explore the information captured through recording movement. Specifically, without the key link between movement patterns and their underlying drivers, the field risks limiting its impact on ecological and evolutionary understanding and its translation to applied objectives (e.g. [84]).

Behavioural valuation of landscapes can greatly enhance our ability to understand mechanisms driving movement patterns, providing insights into classic and emerging topics including cognitive decision-making, memory and the investigation of behavioural tactics manifested in movement. The integration of behavioural theory in movement ecology remains relatively rudimentary, particularly in applied movement ecology—the area where movement analysis is put to conservation needs and uses. This produces general insights but often limits understanding of mechanisms critical for targeted conservation and management action.

Strengthening the behavioural theoretical underpinnings of movement ecological analysis can serve to provide a stronger mechanistic interpretation of movement behaviour and the needs of animals. We conceptualize approaches that translate movement into representations of the behavioural value of locations on landscapes by tracked animals, organizing the broad approaches in movement ecology into four thematic categories of movement-derived metrics: intensity of use, functionality, structural importance and fitness value. We outline the theoretical underpinnings of these metrics to encourage their interpretation from such a foundation. Building from this perspective will ensure the application of each metric to current conservation challenges is mechanistically based. Such valuation enables inference at the local scale emerging from the animal's behaviour (i.e. perspective) that can allow more targeted and efficient management and conservation actions on the landscape. Explicit understanding of why animals use certain locations is critical to reserve design, land-use planning in multi-use landscapes, restoration efforts and determining the impact of natural and human-caused dynamics on species (e.g. climate change). In addition, highlighting the understanding of the movement behaviour itself can garner public and government interest that can be leveraged for conservation returns [85].

Conservation challenges facing natural landscapes are immense. Driven by global food demand, intensification of agriculture and projected increase of 2.3 billion humans [86], 10 million km^2^ are projected to be cleared for agriculture by 2050 [87]. It is imperative that a mechanistic understanding of animal spatial behaviour is leveraged to determine wildlife spatial requirements, and that this information is incorporated in land-use planning efforts. Mechanistic-based valuation of the landscape can provide detailed insights into wildlife needs, focusing conservation efforts at key locations and optimizing investment to critical areas.

## Data accessibility

This article has no additional data.
